# Defining ethical criteria to guide the expanded use of Noninvasive Prenatal Screening (NIPS): Lessons about severity from preimplantation genetic testing

**DOI:** 10.1038/s41431-024-01714-8

**Published:** 2024-10-26

**Authors:** Hortense Gallois, Vardit Ravitsky, Marie-Christine Roy, Anne-Marie Laberge

**Affiliations:** 1 Simon Fraser University, Vancouver, BC Canada; 2 Bioethics, Department of Social and Preventive Medicine, School of Public Health, Université de Montréal, Montreal, QC Canada; 3 President and CEO, The Hastings Center, Garrison, New York USA; 4 Medical Genetics, Department of Pediatrics, CHU Sainte-Justine, Montreal, QC Canada; 5 Department of Pediatrics, Faculty of Medicine, Université de Montréal, Quebec Montreal, Canada

**Keywords:** Ethics, Health policy

## Abstract

We hypothesized that ethical criteria that guide the use of preimplantation genetic testing (PGT) could be used to inform policies about expanded use of non-invasive prenatal screening (NIPS). We used a systematic review of reasons approach to assess ethical criteria used to justify using (or not using) PGT for genetic conditions. Out of 1135 identified documents, we retained and analyzed 216 relevant documents. Results show a clear distinction in acceptability of PGT for medical vs. non-medical conditions. Criteria to decide on use of PGT for medical conditions are largely based on their severity, but there is no clear definition of “severity”. Instead, characteristics of the condition that relate to severity are used as sub-criteria to assess severity. We found that characteristics that are used as sub-criteria for assessing severity include monogenic etiology, high penetrance, absence of treatment, early age of onset, shortened lifespan, and reduced quality of life. Consensus about the use of PGT is highest for conditions that meet most of these criteria. There is no consensus around the acceptability of using PGT to detect non-medical conditions. We propose that the same severity criteria could be used by policymakers to assess the acceptability of using other genetic tests in screening and practice, including for the use of NIPS for additional conditions as indications broaden.

## Introduction

In the late 1990’s, preimplantation genetic testing (PGT) emerged as a revolutionary alternative to invasive prenatal diagnosis [1]. PGT is a technique associated with in vitro fertilization (IVF) that can test embryos for genetic abnormalities. Two decades later, non-invasive prenatal screening (NIPS) was introduced in prenatal care, further changing the face of prenatal screening while raising a new array of ethical implications [2]. NIPS is a more reliable screening technique than other prenatal screening methods (biochemical screening and nuchal translucency measurement), significantly reducing risks of false positives [3]. Like other non-invasive tests, including biochemical screening and nuchal translucency, NIPS is a safe technique compared to invasive diagnostic techniques such as amniocentesis and chorionic villus sampling (CVS). In pregnancies previously identified with a high probability for trisomy 13, 18 and 21, the detection through NIPS is especially accurate. Technology improvements have led NIPS to be used for pregnancies with average levels of probability for trisomy 13, 18 and 21, and for a larger array of conditions. This extended use of NIPS, regardless of a person’s age or other risk factors, was recommended by the ACOG in 2020 [4]. The ability, through constantly improved techniques, to obtain more information on the genetic characteristics of the fetus poses ethical questions. Evaluating the ethical acceptability of various uses of NIPS is of growing importance, especially since private companies are offering the test for an ever-wider range of indications despite relative reliability [5]. The study is part of the PEGASUS-2 project,^1^ which examines the ethical, legal, social, and economic aspects of expanding prenatal screening in Canada using Non-Invasive Prenatal Screening (NIPS). The expanded use of NIPS would involve detecting a broader range of conditions and using NIPS as a first-tier screening tool instead of a second-tier one.

The ethical implications of the use of NIPS are in many ways comparable to those of PGT, as both tools are offered as first-tier genetic tests with the potential to influence the parents’ decision to implant/continue the pregnancy. Like NIPS, PGT is used to provide genetic information without additional risk to the pregnant individual and/or the fetus [6–9]. While both PGT and NIPS are used to assess the risk of genetic abnormalities, NIPS remains a screening tool offered to pregnant individuals, while PGT is a diagnostic tool. Additionally, PGT is more complex and invasive, as it involves testing embryos to select the one(s) to be implanted or not [10–12]. In contrast, NIPS is a less invasive procedure, performed on a blood sample from the pregnant individual and viewed as an aid in decision-making during pregnancy.

Currently, NIPS is primarily used as a screening tool to reduce the need for invasive diagnostic procedures, such as amniocentesis, which carry inherent risks to the pregnancy. However, as genome sequencing techniques become more precise and reliable, the amount and accuracy of information obtained from NIPS is rapidly increasing. The ethical debates surrounding this technology mirror the concerns and implications identified in the literature on PGT [7, 13, 14]. Both techniques raise concerns about the increasing amount of information potentially available from the test, making PGT a relevant proxy for anticipating the range of information that should or should not be sought for NIPS. In addition, both PGT and NIPS reveal a pressing debate about equity in access to prenatal testing/screening technologies, particularly in the context of publicly funded programs. Since the 1990s, an extensive body of literature has developed around the ethical criteria for framing the array of information available through PGT. Reviewing these criteria can help in adopting an anticipatory approach to expanding the use of NIPS. To this end, we conducted a systematic review of the literature to identify the criteria guiding the ethical use of PGT, with the intent of identifying criteria which could also guide the expansion of NIPS beyond the detection of common autosomal aneuploidies.

## Methods

The literature review was based on the methodology of a systematic review of reasons. This type of systematic review is particularly relevant to guide the development and adoption of public health policies. By synthesizing evidence from multiple studies, systematic reviews of reasons can be used to ensure that public health policies are grounded in the best available research, maximizing their effectiveness and efficiency. We followed the Preferred Reporting Items for Systematic Reviews and Meta-Analysis (PRISMA) guidelines [15].

### Search strategy

**Fig. 1:**
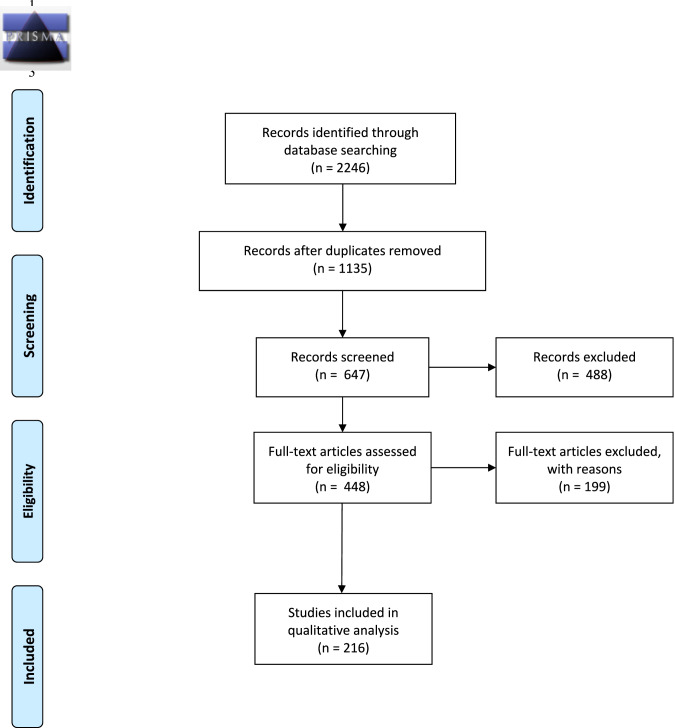
Prisma flow diagram presenting the research and selection of articles. Prisma flow diagram of articles identified, screened, and included for analysis. Eligibility criteria included peer-reviewed and general press articles, book chapters and conference summaries, published in French or English between January 1998 and July 2019 and relating to the ethical criteria for the use of PGT to detect information on the embryo in OECD countries. See methods for further details on screening process and eligibility criteria and supplementary materials for the full list of articles.

We searched four databases (Medline, Embase, Web of Science and Scopus) using key concepts and MeSH descriptors pertaining to our research question (see Table 1 and Supplementary Table 1). The choice of databases was made with a specialized research librarian and aims at providing a broad and balanced coverage of the topic, drawing from diverse disciplines and sources while ensuring high quality and reliability. We used the terms Preimplantation Genetic Diagnosis (PGD) and PGT to cover older and more recent terminology. In this paper, we use the term PGT throughout because it is the currently used terminology. Eligibility criteria for the systematic review included peer-reviewed and general press articles, book chapters and conference summaries, published in French or English between January 1998 and July 2019 and relating to the ethical criteria for the use of PGT to detect information on the embryo in OECD countries. This time frame covers the period since PGT was introduced into practice in the targeted countries. This period also corresponds to the adoption and implementation of public policies for PGT in the targeted countries. Limiting to OECD countries allowed for the inclusion of a diversity of countries and regions where prenatal genetic testing policies are in place, while limiting the results to a reasonable number compared to a global search. Figure 1 presents a PRISMA diagram of the research and the steps for selecting articles.

**Table 1:** Summary of the concepts, keywords and MeSH descriptors selected.

Concept	Genetic testing	Ethical	Prenatal
**Key- word/ MeSH**	Genetic test* Genetic screen* Genetic diagnos*	Ethic* Bioethic*	Embryo In vitro fertilization Preimplantation Fetus Fetal Pregnan* Prenatal Antenatal

Given the large number of results, we decided not to use additional methods such as snowballing. Although designed to cover all genetic prenatal testing techniques, only articles relating to PGT were retained for this review. The rest of the documents will be used in the development of a review of the broader literature.

The choice of PGT is justified by certain similarities in the ethical debate around the criteria for selecting the conditions to test in the context of NIPS and PGT. A comparative table of main characteristics of the use of NIPS, PGT and amniocentesis in Canada is found in supplementary materials (Supplementary Table 2). The challenges of the distinction between NIPS and PGT will be presented in the discussion section.

### Study selection and analysis

Two of the authors (HG and AML) performed the Title and Abstract screening and established a list of eligibility criteria. After reading the body of the texts, one author (HG) selected the relevant documents using the predetermined eligibility criteria defined with AML. Each selected document was analyzed and coded by HG using Nvivo qualitative analysis software, using a node system developed jointly with one co-author (AML). After analyzing and coding the articles, the authors HG and AML identified a specific number of nodes. Each node corresponds to a criterion, reason, or argument related to searching for information using PGT. These nodes were then classified based on how frequently they appeared in the literature.

## Results

The search strategy yielded 2246 documents. After excluding duplicates, 1135 documents were kept for title and abstract screening. The eligibility criteria above were applied by two researchers (HG and AML) after reading the abstracts and titles of all 1135 documents retrieved in the initial phase. Each author individually determined whether the article should be included or not, as well as the reason(s) for inclusion or exclusion. After pooling and discussing the inclusion or exclusion for each article, a consensus was established and eligibility criteria were refined. At this stage, 687 articles were further excluded,^2^ leaving 448 documents for full-text screening. After full-text screening, the final sample included 216 articles. The full list of selected articles can be found in supplementary materials.

The analysis of the articles revealed a clear distinction in acceptability of testing for medical vs. non-medical conditions. Further, arguments in favor of testing for medical conditions were largely based on their severity, while “severity” was not defined per se. However, we identified six disease characteristics related to severity that were used as proxies or sub-criteria assessing severity: the fact that a condition is (1) monogenic, (2) highly penetrant, (3) has no specific treatment, (4) an early age of onset, (5) a shortened lifespan, and (6) implies a reduced quality of life. Out of the 216 articles reviewed, barely any made the claim that testing for non-medical characteristics (especially in the context of a publicly funded testing program), could be justified. We elaborate on these results and the distinction between medical and non-medical conditions below.

### Ethical criteria relating to medical conditions: qualifying severity

Severity is the most cited criterion to justify the use of PGT to detect a genetic condition. “Serious”, “severe” and related terms appeared more than three hundred times in the literature reviewed. The notion of disease severity is also recurrent in public policies and regulations governing the use of PGT. Notably, the Fertilization and Human Embryology Authority (FHEA) in the United Kingdom recommended in a report on the use of PGT that it should be limited to cases of “*risk of a* *serious* *genetic condition in the embryo*” [16] (emphasis ours). The notion of severity is not specific to the literature on PGT [17], but in the context of PGT, the review revealed that severity is often used as the over-arching criterion to distinguish genetic conditions that are ethically acceptable to test for, from those that are less so, or not ethically justified at all [10, 18–32]. Although severity is recurrent in the literature on PGT, no clear definition of the term has emerged [22, 33, 34]. Both the list of genetic conditions qualified as severe and the definition of the concept of severity are far from unanimous in the literature studied [23, 27, 35].

Severity has been linked to defining a threshold from which a life is “worth living” or to support wrongful life or birth claims [10, 27, 31, 36]. PGT is in this sense understood as a form of preventive medicine, the objective being to avoid the creation of a life not worth living because of what is considered to be excessive suffering [27], or as a corollary of the moral duty to do no harm [10]. In this case, some consider it preferable for the child not to be born than to be born to experience a life with pain or suffering, with an extremely shortened lifespan, and/or with severe mental or physical disabilities [37]. There is no consensus on what constitutes undue pain and suffering nor on how or by whom it should be assessed. The question of defining a threshold beyond which life is not worth living is extremely controversial in the literature, and it has been argued that it would be inherently unethical to do so [21].

The detection of “severe” conditions with PGT is also justified by the impact of certain chromosomal abnormalities on the chances of success of a pregnancy. Some aneuploidies are associated with an increased risk of spontaneous abortion. In this case, the ‘severity’ criteria to qualify a condition is relativized by the genetic context of the parents, and their likelihood to conceive. This result seems to indicate that the severity of a condition is perceived differently for different parents, and that the difficulty to procreate related to a condition can increase its severity. The criterion of severity is used in that case to determine what conditions should be detected, as a way to increase the parents’ chances of carrying a pregnancy to term.

Generally, the more severe a disease is considered, the more consensus tends to emerge in the literature on the acceptability of using PGT. Sometimes, severity is presented as the primary criterion to determine the acceptability of testing while secondary sub-criteria can further enhance or decrease the level of acceptability. For example, the 1990 Human Fertilization and Embryology Act^3^ states that licenses for PGT may be given when there is a significant risk that an embryo will have ‘a. a serious physical or mental disability; b. a serious illness; or c. any other serious medical condition’. However, in the case of conditions with lower penetrance, or with later onset, the UK authority considered that further public consultations should be conducted. In this case, severity can justify testing, if the severe condition is also highly penetrant and/or with an early onset [38].

Most often, however, sub-criteria are presented as inherent to the notion of severity, rather than secondary to it [13, 16]. For example, the French National Consultative Committee on Ethics (CCNE; Avis n.107) defined four categories of severe conditions for which the use of PGT is justified. Each category includes different sub-criteria. For instance, one category includes “highly penetrant diseases, with an impact on the quality of life, whether the onset is early (e.g., cystic fibrosis) or later in life (e.g. Huntington for example)” [13]. A disease characterized by most or all of these sub-criteria will more easily generate consensus about acceptability of using PGT. The analysis identified ‘severe’ conditions as typically monogenic and highly penetrant, often lacking specific treatments, shortening lifespan, and having an early onset with a reduced quality of life. Detailed sub-criteria are provided below and summarized in Table 2. Of note, conditions that do not meet these sub-criteria, or only a few, are more likely to create controversy on the use of PGT. Our analysis revealed that conditions which meet some but not all severity sub-criteria tend to fall into an ethical gray zone. The importance given by the author to specific sub-criteria (e.g., age of onset, availability of treatment) will influence the assessment of acceptability.

**Table 2:** Summary of the severity assessment and relationship with the acceptability of PGT for the conditions most frequently cited in the literature^a^.

	Criteria					
	Monogenic	High penetrance (>80%)	Lack of specific treatment	Shortened lifespan	Early onset of symptoms	Reduced quality of life^c^
Condition^b^						
Duchenne muscular dystrophy	+	+	+	+	+	+
Tay Sachs disease	+	+	+	+	+	+
Huntington	+	+	+	+	–	+
Cystic Fibrosis	+	+	+/−	+/−	+	+
Marfan syndrome	+	+	+/−	+/−	+	+/−
Turner syndrome	–	+	+	+/−	+	+/−
Trisomy 21	–	+	+	+/−	+	+/−
Hemophilia	+	+	−	+/−	+	+/−
Gaucher disease	+	+/−	−	+/−	+	+/−
BRCA1/BRCA2-related hereditary cancer syndrome	+	+/−	+/−	+/−	–	+/−
Deafness	−/+	+/−	–	–	+	+/−
Alzheimer’s disease	–	–	–	+/−	–	+
Asthma, eczema	–	–	–	–	+	–

^a^The purpose of the table is to summarize the criteria used to define severity in the literature, and show how they are applied to the conditions most frequently cited in the literature reviewed about PGT.

^b^Conditions in dark gray meet the majority if not all severity criteria, and consensus on acceptability for PGT is high. Conditions in medium gray meet about half of the severity criteria, and there is no clear consensus on acceptability for PGT (acceptability varies from source to source and depends on consideration given to specific criterion). Conditions in light gray meet a minority of severity criteria and consensus on acceptability for PGT is low (rather, high consensus on unacceptability for PGT).

^c^The impact on quality of life is related to the level of physical or psychological suffering considered high and/or to a mental or physical disability that prevents the individual from being independent in adulthood. We have indicated how quality of life is described in the reviewed literature when discussing each specific condition.

#### Monogenic conditions

For complex traits or conditions, which arise from interactions between multiple genes and environmental factors, the use of PGT is generally considered less relevant [27, 39, 40]. Compared to multifactorial equivalents, monogenic forms of a condition often emerge earlier and have a more severe outlook, which could explain why several authors consider justifiable to use PGT [26, 41–43]. Further, the use of PGT for polygenic conditions is not yet integrated into regular clinical practice, reflecting ongoing debates about its accuracy, ethical implications, and clinical utility [41, 44]. The fact that a condition is monogenic also makes it testable by PGT, which can detect its occurrence based on the presence or absence of a given variant. In 2015, the European Society of Reproduction and Embryology Consortium reported the use of PGT for over 190 monogenic conditions in the previous decade [45].

#### High penetrance

The penetrance of a genetic condition refers to the proportion of individuals carrying a genetic variant causing this condition that will develop signs and symptoms [22]. A fully or highly penetrant condition (between 80 and 100%) means that the individual carrying the variant is almost certain to develop the condition in their lifetime. A paradigmatic example of a highly penetrant condition discussed in the literature is Huntington disease^4^ [27, 28, 41, 47]. Originally, PGT was used for highly penetrant conditions almost exclusively [27, 28], but some have argued PGT could ethically be used for conditions with incomplete penetrance (meaning that an individual carrying the variant cannot be sure to develop the associated disease) [27, 41, 48]. Interestingly, arguments in favor of testing for less penetrant disease or even susceptibilities tend to account for other factors of ‘severity’ (e.g. impact on longevity) of these conditions: “we would not consider mild conditions — like asthma and eczema — which can be well managed in medical practice” [[41], p542].

#### Lack of specific treatment

For most genetic conditions, the care of patients is limited to alleviating symptoms, reducing suffering, or improving their overall quality of life, as no treatment exists (neither curative nor preventive). PGT or termination of pregnancy after prenatal diagnosis are in that sense the only options available to avoid the birth of a child who would develop a severe condition. Lack of treatment (or incurability) has been a regulatory criteria justifying PGT in several countries, including Australia [22], France [22, 49, 50], the Netherlands [22, 51], Spain [52], or Sweden [53]. However, some question the ethical acceptability of allocating resources to improving prenatal and preimplantation testing techniques, rather than seeking treatments or improving the care of those affected [54, 55].

#### Shortened lifespan

The impact on longevity is an interesting although not well-defined criteria in the literature. The American Society of Reproductive Medicine (ASRM) has considered that a strong ethical justification for the use of PGT for a condition exists when it “significantly reduces longevity” [[56], p490]. In France, a condition that “prematurely threatens the prospects of life” may trigger the rights to seek PGT (if other conditions apply) [50]. However, the ethical acceptability of PGT for diseases whose prognosis for life expectancy varies widely in affected individuals is less clear, as revealed by debates on the example of cystic fibrosis. While some affected individuals die relatively young, many now live into their forties or fifties [18]. As such, it appears the impact on longevity is a significant sub-criteria, yet it is rarely considered on its own.

#### Early onset of symptoms

Conditions that manifest symptoms particularly early in an individual’s life, either from birth or during childhood, are more easily considered to justify the use of PGT [10, 11, 40, 57]. There are, however, a growing number of late-onset genetic conditions for which PGT is considered desirable [49, 58] based on other severity criteria, such as absence of treatment or impact on quality of life (as in neurodegenerative diseases such as Huntington’s disease, or familial forms of Alzheimer’s disease) [27, 59].

#### Reduced quality of life

Although often referenced, this criterion is probably the most difficult to apprehend, as it relies on both sensitive and subjective factors. The quality of life resulting from a genetic condition is often understood as a reflection of both the level of suffering caused by the condition and its impact on the physical and cognitive abilities of an individual [7, 13, 16, 24]. The latter factor may be associated with the level of autonomy of the individual in adulthood [60]. This factor can be difficult to predict, since certain conditions considered by some as severe have a very variable impact on the level of autonomy, as is the case for Down syndrome [18, 60]. Furthermore, available measures of quality of life have limitations and do not necessarily reflect the perspective of the person living with the condition.

### Ethical debates around non-medical indications

Unlike medical conditions, debates around non-medical indications of PGT lack clear criteria for acceptability and often focus on the child’s best interest. Arguments in favor state that parents have the right to decide what is best for their children, while opposing views emphasize protecting the child’s interests independently of parental views [9, 11, 13, 61–68]. Non-medical information frequently appears in the literature reviewed, highlighting the complexity of these debates. For example, discussions on using PGT to save a sibling consider the severity of the existing child’s condition. While some argue against using PGT for any purpose other than benefiting the prospective child [69, 70], others are concerned with the potential instrumentalization of the child [71–74]. Governments legalizing “savior siblings” often impose severity criteria (although ill-defined) for the condition of the older child [57, 58], and physical and psychological risks for the child created through PGT are carefully weighed against the possibility of saving another child’s life. Additionally, the practice of sampling from the umbilical cord is considered less risky compared to solid organ or bone marrow donation [59].

The issue of sex selection for non-medical reasons is another significant area of debate, addressed by almost half of the reviewed documents. Concerns include reinforcing gender-based discrimination and altering population gender ratios [68, 75–77]. Cultural context plays a crucial role in the acceptability of sex selection, with some justifying it for social reasons like balancing family gender ratios [78], while others fear it might lead to reproductive tourism [5, 58, 79, 80]. Although some argue that parental rights should prevail when no societal impact is evident, others support a global prohibition to prevent discrimination. The use of alternative techniques like sperm sorting, which avoids discarding embryos based on sex, is also discussed [76, 81]. Additionally, promoting the transmission of conditions viewed as disabilities, such as deafness, remains highly controversial, with arguments about reproductive autonomy clashing with concerns about reducing future individuals’ capacities. The selection of “cosmetic” traits through PGT, like eye or hair color, faces strong opposition, even from advocates of a liberal PGT approach [82].

Non-medical information is an essential part of the literature review, and some authors rightfully highlight the lack of clarity regarding what is medical vs. non-medical. As such, non-medical information intersects with questions of severity and the distinction between medical and non-medical categories is evolving and culture laden.

#### Promoting the transmission of a condition

Promoting the transmission of a condition generally viewed by society as a disease or disability through PGT is extremely controversial in the literature reviewed. The most frequent cases are parents living with a condition themselves, wishing to pass on this condition to their child, as a way to ensure their integration into their specific community (e.g., deafness) [47]. Considering it is ethically and legally permissible to search for these specific conditions to prevent the birth of an affected child, some consider that it could be equally possible to search for these conditions to favor their selection [26, 29]. This argument is based on reproductive autonomy arguments and the right of parents to make choices for their child [62]. Others, including the International Bioethics Committee of UNESCO, have argued that such a practice does not take into account the reduced capacities of the future person which impinge unacceptably and irreversibly on their own autonomy [49, 63, 64].

## Discussion

### Defining a decision-making framework using severity

This review aimed at identifying criteria used to determine the ethical considerations framing the use of PGT, with the prospect of identifying criteria which could guide the expansion of NIPS beyond the detection of common autosomal aneuploidies. Our findings show that, although the use of PGT is considered acceptable for the detection of severe genetic conditions, in most cases severity was not explicitly defined. In the past, some jurisdictions have addressed this issue by developing a list of conditions deemed severe enough to justify the use of PGT. The use of a list has been rather limited because it proved impractical: the list is bound to be incomplete and biased in favor of more common conditions. The use of lists therefore raises questions about equity and has been discouraged, including in official public policies [27]. In addition, including a condition in a list of “severe” conditions could ultimately have discriminatory effects on people living with the listed conditions [13].

An alternative to lists of conditions would be the development of a more detailed definition of severity. Such a definition would be useful to guide policymakers, health professionals and prospective parents, as they consider the acceptability of the use of PGT, or other prenatal tests such as NIPS, for a given condition. Our review has led to the identification of condition characteristics that contribute to its perceived severity and are cited frequently in the literature as proxies for severity. Conditions seen as ‘severe’ are characterized as monogenic, highly penetrant, lacking a specific treatment, and having a shortened lifespan, an early age of onset, and reduced quality of life. On this basis, we propose that these characteristics can be used as criteria to assess the overall severity of specific conditions and how conditions compare in terms of severity. Our review does not make it possible to assign weights to these criteria or to come up with a quantitative measure of severity. The importance of each criterion will need to be determined according to the social, cultural, and individual context in which it is embedded.

### Shortcomings of severity

Our review highlighted a problematic lack of consensus around certain terms, including but not limited to severity. Some of the terms commonly used in the reviewed literature refer to concepts whose definition varies widely among authors. Another example is the distinction between “medical” and “non-medical” indications for PGT, for which there are different thresholds and no clear consensus across sources [38]. If we conclude it is ethically more acceptable to search for a condition because we consider it to be a ‘medical’ indication, we may end up “pathologizing” the information parents wish to know [83]. Therefore, caution should be taken when defining what the terms “medical” and “non-medical” encompass.

### Implications for NIPS and ways forward

While PGT and NIPS share some similarities in terms of reliability, PGT is not a screening test. Nevertheless, a low residual risk remains after testing for both PGT and NIPS, for different technical reasons. For this reason, in both cases, it is still recommended to confirm the results through invasive prenatal diagnosis. Additionally, differences between PGT and NIPS limit the application of criteria identified by our review. PGT is generally performed on several embryos at a time, making it possible to choose one that is unaffected for implantation. NIPS is performed during an ongoing pregnancy and the only available choices after confirmation of fetal diagnosis are to continue or terminate the pregnancy. The possible consequences of NIPS thus raise ethical issues that distinguish it from PGT, since termination entails potential physical and psychological harms to the pregnant person and is seen as ethically problematic in many cultures. On the other hand, the destruction of affected embryos during PGT can also raise ethical concerns, particularly in cultures or religious traditions that place significant moral value on embryonic life [11, 34].

Another key difference involves the reason for testing. NIPS may be used purely for information, to allow preparation for the birth of a child with special needs [84]. PGT is very different in this respect, since the purpose of embryo selection is generally to *prevent* the implantation of an affected embryo [85] and hence to prevent the birth of a child with the detected condition (or, in some rare cases as discussed above, to promote the birth of a child with the detected condition, as in the case of a preference for a deaf child). Considering the physical, emotional, and financial burdens associated with in vitro fertilization (IVF; prerequisite to PGT), it is unlikely that prospective parents would undertake PGT purely for information and then randomly implant any embryo. In this context, the indications for NIPS could become much broader than for PGT.

As NIPS progresses technologically and the array of genetic conditions and traits targeted for possible screening grows wider, as is expected, it will be necessary to keep on reflecting on NIPS’ ethical uses. This calls for the development of adequate tools and improvement in the definition of key-terms, starting with severity, as our review suggests.

A clear definition of severity and the use of explicit criteria to determine which conditions are acceptable to test using PGT or NIPS in a particular context are important starting points. The ethical acceptability of investigating a condition is nevertheless not based only on its “objective” severity, but also on individual aspects such as the personal experience and perspective of the parent with the condition or at risk of transmitting it. For that reason, for conditions that meet only some of the severity criteria, the assessment of acceptability may still require individual case-by-case analysis. This approach should involve comprehensive discussions with the prospective parent(s) to ensure informed consent, including detailed information about the risks, benefits, and limitations of the test, particularly for conditions for which accuracy remains limited [20, 33, 34, 40, 44, 85].

## Conclusion

It has been “shown that it is technically feasible to sequence the entire fetal genome using [NIPS], although this is not yet achievable in a timely or cost-effective way” [[86], p10]. Like with PGT, the increasing array of genetic information potentially available calls for the adoption of clear ethical criteria to determine what information it is justified to seek.

Beyond the question of the amount of information accessible by the test, issues of equity in access to the test itself raise significant questions that any public healthcare system should consider [37, 40]. Furthermore, since PGT is only performed in a limited number of pregnancies and mostly privately funded, a reliance on case-by-case analysis is feasible. By contrast, NIPS is already implemented in several publicly funded healthcare systems as part of prenatal screening programs for aneuploidy. A case-by-case analysis of the acceptability of the use of NIPS for other conditions is not realistic considering its vast use. The application of recognized criteria will be necessary to determine at the healthcare system level whether adding specific conditions to the NIPS panel is ethically acceptable. Hence, improving the understanding of key terms such as severity, quality of life, and what constitutes medical vs. non-medical indications, would be beneficial to help policymakers, healthcare professionals and patients in their decisions about the use and coverage of NIPS and other forms of reproductive testing. The review highlights the pivotal yet ambiguously defined role of “severity” and “seriousness” in guiding legal, ethical, and clinical decisions regarding PGT across various jurisdictions. Although this problematic ambiguity had gained attention [87], further concerted interdisciplinary research and action by policymakers is needed to refine and clearly define the concept of severity, especially in light of the rapid advancements in non-invasive prenatal screening (NIPS).

## Supplementary information

Supplementary Tables

Annex - List of titles

## Footnotes

1 For more information on the project, see https://genomecanada.ca/project/pegasus-2-personalized-genomics-prenatal-abnormalities-screening-using-maternal/.

2 The exclusion criteria established at this stage made it possible to exclude technical articles relating to prenatal tests without ethical considerations; surveys or interviews reporting personal opinions or attitudes towards prenatal tests (both among health professionals, patients and their relatives or the general population) without ethical argumentation on the test criteria (i.e., studies on fears regarding to the implementation of the test in the clinical context); documents relating to the reasons leading pregnant people or couples to request the test; articles relating to the clinical and technical aspects of using the test (i.e. articles describing the ethical aspects of obtaining patient consent); articles listing the laws or regulations in place without argumentation on the ethical reasons leading to their adoption.

3 Of note, the 1990 HFEA was not a document identified in this literature review, since it is out of the research scope (1998–2019). The Act is cited as part of the argument made by Bayefski, 2017 [17], which is part of the review.

4 The penetrance of Huntington disease is determined by the number of CAG repeats in the HTT gene. Huntington disease shows full penetrance in individuals with 40 or more CAG repeats in the HTT gene, while 36–39 repeats result in reduced penetrance, meaning some individuals may remain asymptomatic [46].
