# Supplementary material for: Defining ethical criteria to guide the expanded use of Noninvasive Prenatal Screening (NIPS): Lessons about severity from preimplantation genetic testing
Source: Eur J Hum Genet. 2024 Oct 26;33(2):167–75. doi: 10.1038/s41431-024-01714-8 (PMC11840150; doi:10.1038/s41431-024-01714-8)
Supplement: Supplementary file 1 — Supplementary Tables [file 41431_2024_1714_MOESM1_ESM.docx]

***Supplementary Materials***

**Supplementary Table 1: Research Equation Used on Medline**

| 1. exp ETHICS/ |
| --- |
| 2. (ethic* or bioethic*).ab,kf,kw,ti. |
| 3. 1 or 2 |
| 4. Genetic Testing/ |
| 5. Preimplantation Diagnosis/ |
| 6. exp Prenatal Diagnosis/ |
| 7. (non invasive prenatal test or non invasive prenatal screening).ab,kf,kw,ti. |
| 8. NIPT.ab,kf,kw,ti. |
| 9. ((prenatal* or antenatal* or genetic*) adj3 (screen* or test* or diagnos*)).ab,kf,kw,ti. |
| 10. 4 or 5 or 6 or 7 or 8 or 9 |
| 11. embryonic structures/ or embryo, mammalian/ |
| 12. Fetus/ |
| 13. (fetus* or prenatal* or antenatal* or embryo* or preimplantion* or pregnan*).ab,kf,kw,ti. |
| 14. 11 or 12 or 13 |
| 15. 3 and 10 and 14 |
| 16. (Australia or Austria or Belgium or Canada or Chile or Czech Republic or Denmark or Estonia or Finland or France or Germany or Greece or Hungary or Iceland or Ireland or Israel or Italy or Japan or Korea or Luxembourg or Mexico or Netherlands or New Zealand or Norway or Poland or Portugal or Slovak Republic or Slovenia or Spain or Sweden or Switzerland or Turkey or United Kingdom or England or Wales or Scotland or United  States).ab,kf,kw,ti. |
| 17. Australia/ or Austria/ or Belgium/ or Canada/ or Chile/ or Czech Republic/ or Denmark/ or Estonia/ or Finland/ or France/ or Germany/ or Greece/ or Hungary/ or Iceland/ or Ireland/ or Israel/ or Italy/ or Japan/ or Korea/ or Luxembourg/ or Mexico/ or Netherlands/ or New Zealand/ or Norway/ or Poland/ or Portugal/ or Slovak Republic/ or Slovenia/ or Spain/ or Sweden/ or Switzerland/ or Turkey/ or United Kingdom/ or England/ or Wales/ or Scotland/ or  United State |
| 18. 16 or 17 |
| 19. 15 and 18 |
| 20. limit 19 to yr="1998 -Current" |

**Supplementary Table 2: Comparative Table of Main Aspects Related to the Use of NIPS, PGT and Amniocentesis in Canada**

| **Test**  **Aspect** | **PGT** | **NIPS** | **Amniocentesis** |
| --- | --- | --- | --- |
| Subject | Several embyros pre-implantation | One fetus | One fetus |
| Time | Day 3 to 5 after IVF | From the 10th week of pregnancy | From the 16th week of pregnancy |
| Reliability | Diagnostic | Screening | Diagnostic |
| Procedure | Biopsy and genetic tests | Blood sample from the parent and genetic tests from  fetal cells | Amniotic fluid collection and genetic testing |
| Invasiveness | Yes | No | Yes |
| Cost | High | Relatively low | High |
| Covering | Private | Private except for high-risk pregnancies in Ontario, British Colombia, Quebec, Nova Scotia, Yukon | Public with prescription |
| Physical risks | No physical risk for the parent apart from constraints of IVF | No physical risk for the parent | Risks of spontaneous abortion and complication |
| Main reasons for testing | - Known risk of genetic or hereditary abnormality - Infertility or repeated spontaneous abortions | High risk of trisomy 13, 18, 21 revealed by a first-line test | High risk of chromosome abnormality revealed by a first-line test, a family history or ultrasound abnormalities |
| Possible outcome | - Selection of one (or more) healthy embryos and implantation - Selection of one (or more) healthy embryos and conservation for the future - No follow-up (no healthy embryo and / or abandonment of the parental project) | Informing the parent of the presence or absence of a probability that the fetus will be affected followed:  - Use of an invasive diagnostic test  - Continuation of pregnancy without additional testing  - Termination of pregnancy without additional testing | Informing the parent of the presence or absence of an abnormality in the fetus:  - Continuation of pregnancy   - Termination of pregnancy |
| Information currently sought in view of the risks, constraints and costs | - Chromosomal anomalies  - Monogenic diseases  - Sex (medical reasons)  - Compatibility status with a sibling  Selection based on other criteria determined by parent | - T13, 18 and 21  - Sex chromosome aneuploidies  - Microdeletions | - Chromosomal abnormality   - Monogenic disease |
