# Supplementary material for: Defining ethical criteria to guide the expanded use of Noninvasive Prenatal Screening (NIPS): Lessons about severity from preimplantation genetic testing
Source: Eur J Hum Genet. 2024 Oct 26;33(2):167–75. doi: 10.1038/s41431-024-01714-8 (PMC11840150; doi:10.1038/s41431-024-01714-8)
Supplement: Supplementary file 2 — Annex - List of titles [file 41431_2024_1714_MOESM2_ESM.docx]

**Annex: Full list of references included in the review in chronological order (N=216)**

| **Year Range** | **Items** | **%** |
| --- | --- | --- |
| 2016-2019 | 17 | 7.9 |
| 2011-2015 | 36 | 16.7 |
| 2006-2010 | 56 | 25.9 |
| 2001-2005 | 94 | 43.5 |
| 1998-2000 | 13 | 6.0 |
| ***Total (1998-2019)*** | ***216*** |  |

1. Hens K, Dondorp W, De Wert G. Blurring boundaries. Interviews with PGT couples about comprehensive chromosome screening. *European Journal of Medical Genetics*. Published online 2019.

2. Baertschi B. Preimplantation genetic diagnosis (PGD) in the era of predictive medicine. *Med Sci (Paris)*. 2019;35(1):72-77. doi:10.1051/medsci/2018292

3. Zielinska AC, Lowy I. The Golden Helix: Origins, ethnicity and preconception genetic screening in Israel. *BioSocieties*. 2018;13(2):323-348.

4. Takahashi S, Patrizio P. Shall we “Pause” PGS? *Journal of Obstetrics and Gynaecology Research*. 2018;44(8):1648.

5. Smith MK, Taylor-Sands M. Comparing Non-Medical Sex Selection and Saviour Sibling Selection in the Case of JS and LS v Patient Review Panel: Beyond the Welfare of the Child? *Journal of Bioethical Inquiry*. 2018;15(1):139-153.

6. Hashiloni-Dolev Y. The effect of Jewish-Israeli family ideology on policy regarding reproductive technologies. In: *Bioethics and Biopolitics in Israel: Socio-Legal, Political, and Empirical Analysis*. Cambridge University Press; 2018:119-138.

7. Farrell RM, Allyse MA. Key Ethical Issues in Prenatal Genetics: An Overview. *Obstet Gynecol Clin North Am*. 2018;45(1):127-141. doi:10.1016/j.ogc.2017.10.006

8. Zuckerman S, et al. Acceptable applications of preimplantation genetic diagnosis (PGD) among Israeli PGD users. *European Journal of Human Genetics*. 2017;25(10):1113-1117.

9. Thompson C. Human embryos: Collect reliable data on embryo selection. *Nature*. 2017;551(7678):33.

10. Frati P, Fineschi V, Di Sanzo M, La Russa R, Scopetti M, Severi FM, et al. Preimplantation and prenatal diagnosis, wrongful birth and wrongful life: a global view of bioethical and legal controversies. *Hum Reprod Update*. 2017;23(3):338-357. doi:10.1093/humupd/dmx002

11. Duguet AM, Boyer-Beviere B. Preimplantation Genetic Diagnosis: The Situation in France and in Other European Countries. *Eur J Health Law*. 2017;24(2):160-174. doi:10.1163/15718093-12420347

12. Bayefsky MJ. Comparative preimplantation genetic diagnosis policy in Europe and the USA and its implications for reproductive tourism. *Reprod Biomed Soc Online*. 2017;3:41-47. doi:10.1016/j.rbms.2017.01.001

13. Sueoka K. Preimplantation genetic diagnosis: An update on current technologies and ethical considerations. *Reproductive Medicine and Biology*. 2016;15(2):69-75.

14. Schoen U, Muller CR, Evers C, Kunz C, Guntinas-Lichius O. Pre-implantation Genetic Diagnosis (PGD) Center, MGZ Munich. *Medizinische Genetik*. 2016;28(1):166-167.

15. Riezzo I, Neri M, Bello S, Pomara C, Turillazzi E. Italian law on medically assisted reproduction: do women’s autonomy and health matter? *BMC Women’s Health*. 2016;16:44.

16. Iltis AS. Prenatal screening and prenatal diagnosis: Contemporary practices in light of the past. *Journal of Medical Ethics*. 2016;42(6):334-339.

17. Fovargue S, Bennett R. WHAT ROLE SHOULD PUBLIC OPINION PLAY IN ETHICO-LEGAL DECISION MAKING? THE EXAMPLE OF SELECTING SEX FOR NON-MEDICAL REASONS USING PREIMPLANTATION GENETIC DIAGNOSIS. *Medical Law Review*. 2016;24(1):34-58. doi:10.1093/medlaw/fwv046

18. Taylor-Sands M. Summary of saviour siblings. *Journal of Medical Ethics*. 2015;41(12):926.

19. Sato K, Yoshimura Y, Wagatsuma H, Yano T, Ishizuka B. Current status of preimplantation genetic diagnosis in Japan. *Bioinformation*. 2015;11(5):254-260.

20. Plichtová J, Moulin-Doos C. Discourses on medical interventions in human reproduction (PGD and ART), state interventions and their justifications: Comparison of Slovak and German cases. *Human Affairs*. 2015;25(2):204-229.

21. Ouellette A. Selection against Disability: Abortion, ART, and Access. *The Journal of law, medicine & ethics: a journal of the American Society of Law, Medicine & Ethics*. 2015;43(2):211-223.

22. Kim NK. Legislation on Genetic Diagnosis: Comparison of South Korea and Germany: - With Focus on the Application and Communication Structure. *Balsaenggwa Saengsig*. 2015;19(2):111-118.

23. Kiefer B. New law on medically assisted procreation: A necessary do-it-yourself. *Revue Medicale Suisse*. 2015;11(474):1096.

24. Gonçalves B. [RESEARCHES ON EMBRYO: THE RISKS OF EUGENIC DRIFTS AND LIVING MATTER UNDER PATENT]. *J Int Bioethique Ethique Sci*. 2015;26(3):167-183, 268-269.

25. Galst JP, Verp MS. *Prenatal and Preimplantation Diagnosis: The Burden of Choice*. Springer; 2015.

26. Ethics Committee of the American Society for Reproductive Medicine. Use of reproductive technology for sex selection for nonmedical reasons. *Fertil Steril*. 2015;103(6):1418-1422. doi:10.1016/j.fertnstert.2015.03.035

27. Breuning M, Bolt I, Bredenoord A, Galjaard RJ, Giltay J, van Lith J et al. Pre-implantation genetic diagnosis in the Netherlands: Experience of a national committee assessing disease eligibility. *Prenatal Diagnosis*. 2015;S1:69-70.

28. Bayefsky M. The regulatory gap for preimplantation genetic diagnosis. *Hastings Cent Rep*. 2015;45(1):7-8. doi:10.1002/hast.412

29. Arzheimer K. Strange bedfellows: the Bundestag’s free vote on pre-implantation genetic diagnosis (PGD) reveals how Germany’s restrictive bioethics legislation is shaped by a Christian Democratic/New Left issue-coalition. *Research & Politics*. 2015;2(3):2053168015601130. doi:10.1177/2053168015601130

30. Valongo A. Human rights and reproductive choices in the case-law of Italian and European courts. *European Journal of Health Law*. 2014;21(2):123-140.

31. Leiter G. What Israeli policy can teach us about elective sex selection. *Israel Journal of Health Policy Research*. 2014;3:3.

32. Dagan E, Gershoni-Baruch R, Kurolap A, Goldberg Y, Fried G. “I do not want my baby to suffer as I did”; prenatal and preimplantation genetic diagnosis for BRCA1/2 mutations: a case report and genetic counseling considerations. *Genet Test Mol Biomarkers*. 2014;18(7):461-466. doi:10.1089/gtmb.2013.0513

33. Scott R. Reconsidering “wrongful life”? in England after thirty years: Legislative mistakes and unjustifiable anomalies. *The Cambridge Law Journal*. 2013;72(1):115-154.

34. Kovacs G. Should Couples Be Allowed to Select the Sex of Their Offspring? *Journal of Obstetrics and Gynaecology Canada*. 2013;35(12):1105-1107.

35. Hens K, Dondorp W, Handyside AH, Harper J, Newson AJ, Pennings G, et al. Dynamics and ethics of comprehensive preimplantation genetic testing: A review of the challenges. *Human Reproduction Update*. 2013;19(4):366-375.

36. Hens K, Dondorp WJ, Geraedts JPM, de Wert GM. Comprehensive embryo testing. Experts’ opinions regarding future directions: An expert panel study on comprehensive embryo testing. *Human Reproduction*. 2013;28(5):1418-1425.

37. Walters L. Genetics and bioethics: How our thinking has changed since 1969. *Theoretical medicine and bioethics*. 2012;33(1):83-95.

38. Pavone V, Arias F. Beyond the Geneticization Thesis: The Political Economy of PGD/PGS in Spain. *Science Technology & Human Values*. 2012;37(3):235-261.

39. Nau JY. Eradiquer les trisomiques, bien qu’ils soient doux (2). *Rev Med Suisse*. 2012;338(16):918-919.

40. Nau JY. Eradicate trisomics, although they are mild (2). *Revue Medicale Suisse*. 2012;8(338):918-919.

41. Malek J, Daar J. The Case for a Parental Duty to Use Preimplantation Genetic Diagnosis for Medical Benefit. *American Journal of Bioethics*. 2012;12(4):3-11.

42. Lutz EEV. Preimplantation genetic diagnosis (PGD) according to medical ethics and medical law. *Journal of the Turkish German Gynecology Association*. 2012;13(1):50-55.

43. Lacombe D. Chapter 9. Medical genetics and ethics. *Journal International de Bioethique*. 2012;32:95-102.

44. LaBonte ML. An analysis of US fertility centre educational materials suggests that informed consent for preimplantation genetic diagnosis may be inadequate. *Journal of Medical Ethics*. 2012;38(8):479-484.

45. Coron F, Rousseau T, Jondeau G, Gautier E, Binquet C, Gouya L, et al. What do French patients and geneticists think about prenatal and preimplantation diagnoses in Marfan syndrome? *Prenat Diagn*. 2012;32(13):1318-1323. doi:10.1002/pd.4008

46. Appel JM. Toward an ethical eugenics: the case for mandatory preimplantation genetic selection. *JONAS Healthc Law Ethics Regul*. 2012;14(1):7-13. doi:10.1097/NHL.0b013e318244c69b

47. Aghajanova L, Valdes CT. Sex Selection for Nonhealth-Related Reasons. *AMA Journal of Ethics*. 2012;14(2):105-111. doi:10.1001/virtualmentor.2012.14.2.ccas3-1202

48. Whittaker AM. Reproduction opportunists in the new global sex trade: PGD and non-medical sex selection. *Reproductive Biomedicine Online*. 2011;23(5):609-617.

49. Valkenburg G, Aarden E. Constructing embryos, constructing politics: Connecting politics and technology in the Netherlands and Germany. *BioSocieties*. 2011;6(4):447-465.

50. Madanamoothoo A. Saviour-sibling and the psychological, ethical and judicial issues that it creates: Should English and French legislators close the Pandora’s box? *European Journal of Health Law*. 2011;18(3):293-303.

51. Krahn TM. Regulating preimplantation genetic diagnosis: The case of down’s syndrome. *Medical Law Review*. 2011;19(2):157-191.

52. Dondorp W, de Wert G. The categorical ban on sex selection for non-medical reasons is in need of urgent reconsideration. *Human Reproduction*. 2011;26(suppl_1):i223-i226. doi:10.1093/humrep/26.s1.81

53. Brandenburg K. Risk, parental autonomy and the epistemic divide: preimplantation genetic diagnosis in the Australian print news media, 1990–2007. *New Genetics and Society*. 2011;30(1):115-131. doi:10.1080/14636778.2011.556710

54. Strange H. Non-medical sex selection: Ethical issues. *British Medical Bulletin*. 2010;94(1):7-20.

55. Sleeboom-Faulkner M. Reproductive technologies and the quality of offspring in Asia: reproductive pioneering and moral pragmatism? *Culture Health & Sexuality*. 2010;12(2):139-152.

56. Shah KR. Selecting barrenness: The use of preimplantation genetic diagnosis by congenitally infertile women to select for infertility. *Human Reproduction and Genetic Ethics*. 2010;16(1):7-21.

57. Macklin R. The ethics of sex selection and family balancing. *Seminars in Reproductive Medicine*. 2010;28(4):315-321.

58. Le Coz P. Preimplantation genetic diagnosis: The recommendations of French Committee of Ethics. *Reproductive Biomedicine Online*. 2010;1:S12.

59. Kent A. Prenatal diagnostics: How to define the boundaries of ethical practice? (A patient and family perspective on current issues and possibilities). *Expert Opinion on Medical Diagnostics*. 2010;4(2):99-101.

60. Hershberger PE, Pierce PF. Conceptualizing couples’ decision making in PGD: Emerging cognitive, emotional, and moral dimensions. *Patient Education and Counseling*. 2010;81(1):53-62.

61. Drury KC. Integrating Preimplantation Genetic Diagnosis into the ART Laboratory. In: Carrell DT, Peterson CM, eds. *Reproductive Endocrinology and Infertility: Integrating Modern Clinical and Laboratory Practice*. Springer; 2010:647-661. doi:10.1007/978-1-4419-1436-1_43

62. Camporesi S. Choosing Deafness with Preimplantation Genetic Diagnosis: An Ethical Way to Carry on a Cultural Bloodline? *Cambridge Quarterly of Healthcare Ethics*. 2010;19(1):86-96. doi:10.1017/S0963180109990272

63. Bouffard C, Godin JK, Bévière B. State Intervention in Couples’ Reproductive Decisions: Socioethical Reflections Based on the Practice of Preimplantation Genetic Diagnosis in France. *AJOB Primary Research*. Published online September 17, 2010. Accessed May 19, 2024. https://www.tandfonline.com/doi/abs/10.1080/21507716.2010.505897

64. Bonneau D, Marlin S, Sanlaville D, Dupont JM, Sobol H, Gonzales M, et al. [Genetic testing in the context of the revision of the French law on bioethics]. *Pathologie-biologie*. 2010;58:396-401.

65. Strong KA. Informing patients about emerging treatment options: Creating “saviour siblings” for haemopoietic stem cell transplant. *Medical Journal of Australia*. 2009;190(9):506-507.

66. Ricci ML. Assisted procreation and its relationship to genetics and eugenics. *Human Reproduction and Genetic Ethics*. 2009;15(1):7-27.

67. Krahn T. Preimplantation genetic diagnosis: Does age of onset matter (anymore)? *Medicine, Health Care and Philosophy*. 2009;12(2):187-202.

68. Jones DG, Hunter KA, Keenan JM, Stewart CR. Who gets born? How did New Zealand’s bioethics council arrive at its recommendations? *New Zealand Medical Journal*. 2009;122(1294):84-91.

69. Gutiérrez-Mateo C, Sánchez-García JF, Fischer J, Tormasi S, cohen J, Munne S, et al. Preimplantation genetic diagnosis of single-gene disorders: experience with more than 200 cycles conducted by a reference laboratory in the United States. *Fertil Steril*. 2009;92(5):1544-1556. doi:10.1016/j.fertnstert.2008.08.111

70. Fortuny D, Balmaña J, Graña B, Torres A, Ramon y Cajal T, Darder E, et al. Opinion about reproductive decision making among individuals undergoing BRCA1/2 genetic testing in a multicentre Spanish cohort. *Hum Reprod*. 2009;24(4):1000-1006. doi:10.1093/humrep/den471

71. Farrelly C. Preimplantation genetic diagnosis, reproductive freedom, and deliberative democracy. *J Med Philos*. 2009;34(2):135-154. doi:10.1093/jmp/jhp016

72. Colls P, Silver L, Olivera G, Weier J, Escudero T, Goodall N, et al. Preimplantation genetic diagnosis for gender selection in the USA. *Reprod Biomed Online*. 2009;19 Suppl 2:16-22. doi:10.1016/s1472-6483(10)60273-8

73. Burton A. Controversy surrounds the selection of embryos to avoid cancer. *Lancet Oncol*. 2009;10(6):545. doi:10.1016/s1470-2045(09)70150-0

74. Turillazzi E, Fineschi V. Preimplantation genetic diagnosis: A step by step guide to recent Italian ethical and legislative troubles. *Journal of Medical Ethics*. 2008;34(10).

75. Snelling J. Embryonic HLA tissue typing and made-to-match siblings: The New Zealand position. *Medical Law International*. 2008;9(1):13-43.

76. Savell K, Karpin I. The meaning of “serious disability” in the legal regulation of prenatal and neonatal decision-making. *Journal of law and medicine*. 2008;16(2):233-245.

77. Landau R. Sex selection for social purposes in Israel: Quest for the “perfect child” of a particular gender or centuries old prejudice against women? *Journal of Medical Ethics*. 2008;34(9).

78. Klitzman R, Appelbaum PS, Chung WK, Sauer MV. Anticipating issues related to increasing preimplantation genetic diagnosis use: A research agenda. *Reproductive Biomedicine Online*. 2008;17(SUPPL. 1):33-42.

79. Hansson SO. Three bioethical debates in Sweden. *Cambridge Quarterly of Healthcare Ethics*. 2008;17(3):261-269.

80. Grazi RV, Wolowelsky JB, Krieger DJ. Sex selection by preimplantation genetic diagnosis (PGD) for nonmedical reasons in contemporary Israeli regulations. *Camb Q Healthc Ethics*. 2008;17(3):293-299. doi:10.1017/S0963180108080353

81. Byk C. Preimplantation genetic diagnosis: an ambiguous legal status for an ambiguous medical and social practice. *J Int Bioethique*. 2008;19(3):87-104, 125. doi:10.3917/jib.193.0087

82. Asscher ECA. The regulation of preimplantation genetic diagnosis (PGD) in the Netherlands and the UK: a comparative study of the regulatory frameworks and outcomes for PGD. *Clinical Ethics*. 2008;3(4):176-179. doi:10.1258/ce.2008.008036

83. Williams C, Ehrich K, Farsides B, Scott R. Facilitating choice, framing choice: Staff views on widening the scope of preimplantation genetic diagnosis in the UK. *Social Science and Medicine*. 2007;65(6):1094-1105.

84. Scott R, Williams C, Ehrich K, Farsides B. The appropriate extent of pre-implantation genetic diagnosis: Health professionals’ and scientists’ views on the requirement for a “significant risk of a serious genetic condition.” *Medical Law Review*. 2007;15(3):320-356.

85. Liu CK. “Saviour Siblings”? The distinction between PGD with HLA tissue typing and preimplantation HLA tissue typing. *Journal of Bioethical Inquiry*. 2007;4(1):65-70.

86. Karpin I. Choosing disability: preimplantation genetic diagnosis and negative enhancement. *Journal of law and medicine*. 2007;15(1):89-102.

87. Kaebnick GE. Liberty and solidarity. *Hastings Center Report*. 2007;37(4):2.

88. Hashiloni-Dolev Y, Shkedi S. On new reproductive technologies and family ethics: Pre-implantation genetic diagnosis for sibling donor in Israel and Germany. *Social Science and Medicine*. 2007;65(10):2081-2092.

89. Borkenhagen A, Brähler E, Wisch S, Stöbel-Richter Y, Strauss B, Kentenich H. Attitudes of German infertile couples towards preimplantation genetic diagnosis for different uses: a comparison to international studies. *Hum Reprod*. 2007;22(7):2051-2057. doi:10.1093/humrep/dem110

90. Williams C. Dilemmas in fetal medicine: Premature application of technology or responding to women’s choice? *Sociology of Health and Illness*. 2006;28(1):1-20.

91. Whelan J. Sex is for fun, IVF is for children. *New Scientist*. 2006;192(2574):42-45.

92. Weiss R. Human embryos in Britain may be screened for cancer risk. *Washington Post*. 2006.

93. Thomas C. Preimplantation genetic diagnosis: Development and regulation. *Medicine and Law*. 2006;25(2):365-378.

94. Snelling J. Implications for providers and patients: A comment on the regulatory framework for preimplantation genetic diagnosis in New Zealand. *Medical Law International*. 2006;8(1):23-49.

95. Scott R. Choosing between possible lives: Legal and ethical issues in preimplantation genetic diagnosis. *Oxford Journal of Legal Studies*. 2006;26(1):153-178.

96. Rothman BK. Consequences of sex selection. *The Chronicle of higher education*. 2006;52(25).

97. Ram NR. Britain’s new preimplantation tissue typing policy: an ethical defence. *Journal of Medical Ethics*. 2006;32(5):278-282.

98. Offit K, Thom P, Hurley K, Filippini K, Schrag D. Cancer genetic testing and assisted reproduction. *Journal of Clinical Oncology*. 2006;24(29):4775-4782.

99. Nelson EL. Comparative perspectives: regulating preimplantation genetic diagnosis in Canada and the United Kingdom. *Fertility and Sterility*. 2006;85(6):1646-1652.

100. Nau JY. Sur la frontière d’un nouvel eugénisme. *Rev Med Suisse*. 2006;082:2314-2314.

101. Knoppers BM, Bordet S, Isasi RM. Preimplantation genetic diagnosis: An overview of socio-ethical and legal considerations. *Annual Review of Genomics and Human Genetics*. 2006;7:201-221.

102. Harris J. The moral choice: prospective mothers should have the right to select embryos on the basis of knowing as much as possible about their future health. *New Scientist*. 2006:24.

103. Harmon A. Couples cull embryos to halt heritage of cancer. *The New York Times*. 2006.

104. Frassoni F. The laws covering in vitro fertilization and embryo research in Italy. *Bone Marrow Transplant*. 2006;38(1):5-6. doi:10.1038/sj.bmt.1705387

105. Finck C, Meister U, Stöbel-Richter Y, Borkenhagen A, Brähler E. Ambivalent attitudes towards pre-implantation genetic diagnosis in Germany. *Eur J Obstet Gynecol Reprod Biol*. 2006;126(2):217-225. doi:10.1016/j.ejogrb.2005.11.031

106. Feyereisen E, Frydman N. [Preimplantation genetic diagnosis]. *Rev Prat*. 2006;56(5):513-519.

107. Duke K. Belgian loophole allows Swiss parents a “saviour” baby. *The Lancet*. 2006;368(9533):355-356. doi:10.1016/S0140-6736(06)69089-2

108. Dresser R. Preimplantation genetic diagnosis as medical innovation: reflections from The President’s Council on Bioethics. *Fertil Steril*. 2006;85(6):1633-1637. doi:10.1016/j.fertnstert.2006.01.013

109. Braude Peter. Preimplantation Diagnosis for Genetic Susceptibility. *New England Journal of Medicine*. 2006;355(6):541-543. doi:10.1056/NEJMp068139

110. Regulating preimplantation genetic diagnosis: the pathologization problem. *Harv Law Rev*. 2005;118(8):2770-2791.

111. Van Balen F. The choice for sons or daughters. *Journal of Psychosomatic Obstetrics and Gynecology*. 2005;26(4):229-230.

112. Tilstone C. UK clinicians to screen embryos for BRCA mutations. *Lancet Oncology*. 2005;6(6):358.

113. Thornhill C. Embryo screening approved for retinoblastoma. *Lancet Oncology*. 2005;6(10):742.

114. Steffann J, Feyereisen E, Kerbrat V, Romana S, Frydman N. [Prenatal and preimplantation genetic diagnosis: decision tree, new practices?]. *M S-Medecine Sciences*. 2005;21(11):987-992.

115. Steffann J, Frydman N, Burlet P, Gigarel N, Feyereisen E, Kerbrat V, et al. Extending preimplantation genetic diagnosis to HLA typing: The Paris experience. *Gynecologie Obstetrique et Fertilite*. 2005;33(10):824-827.

116. Simonstein F. Genetics screening and reproductive choice: Is making a child to save another unethical? *Medicine and Law*. 2005;24(4):775-781.

117. Neurology TL. Screening for disease: how far is too far? *The Lancet Neurology*. 2005;4(1):1. doi:10.1016/S1474-4422(04)00941-X

118. McLean SAM. Sex selection: Intergenerational justice or injustice? *Medicine and law*. 2005;24(4):761-773.

119. Mackenzie R. Reprogenetics and pharmacogenetics: in whose best interests? *Medicine & Law*. 2005;24(2):343-354.

120. Levine AD. A case for government-sponsored monitoring of preimplantation genetic diagnosis in the United States. *Journal of Public and International Affairs*. 2005;16:26-45.

121. Lebacqz K. Choosing our children the uneasy alliance of law and ethics in John Robertson’s thought. *Expanding Horizons in Bioethics, Springer Netherlands*. Published online 2005:123-139.

122. Kahraman S. Are we talking about the butterflies or a butterfy effect?: Comment on “No country is an island.” *Reproductive Biomedicine Online*. 2005;11(1):14-15.

123. Ilencikova D. Clinical genetics: views of ethical and legislative conclusions in the Slovak Republic. *Human Reproduction and Genetic Ethics*. 2005;11(2):49-54.

124. Hudson K, Scott J, Faden R, Basch R. Genetic testing of human embryos ethical challenges and policy choices. *Expanding Horizons in Bioethics, Springer Netherlands*. Published online 2005:103-122.

125. House of Commons S, Technology. Human reproductive technologies and the law: a select committee report. *Bulletin of Medical Ethics*. 2005;(208):13-21.

126. Hocking BA, Guy S. Desperately seeking donors: the “saviour sibling” decision in Quintavalle v Human Fertilisation and Embryology Authority (UK). *Australian Journal of Family Law*. 2005;19(2):144-152.

127. Galton DJ. Eugenics: some lessons from the past. *Reprod Biomed Online*. 2005;10 Suppl 1:133-136. doi:10.1016/s1472-6483(10)62222-5

128. Fagniez PL, Loriau J, Tayar C. ["Designer baby" changed to French for “double hope baby”]. *Gynecol Obstet Fertil*. 2005;33(10):828-832. doi:10.1016/j.gyobfe.2005.07.034

129. Downing KK. A feminist is a person who answers “yes” to the question, “are women human?”: an argument against the use of preimplantation genetic diagnosis for gender selection. *DePaul J Health Care Law*. 2005;8(2):431-460.

130. Dickens BM. Preimplantation genetic diagnosis and “savior siblings.” *Int J Gynaecol Obstet*. 2005;88(1):91-96. doi:10.1016/j.ijgo.2004.10.002

131. Dahl E. No country is an island: comment on the House of Commons report Human Reproductive Technologies and the Law. *Reproductive BioMedicine Online*. 2005;11(1):10-11. doi:10.1016/S1472-6483(10)61288-6

132. Cameron NM de S. Pandora’s progeny: ethical issues in assisted human reproduction. *Fam Law Q*. 2005;39(3):745-779.

133. Bennett B. Symbiotic relationships: saviour siblings, family rights and biomedicine. *Aust J Fam Law*. 2005;19(3):195-212.

134. Bellamy S. Lives to save lives--the ethics of tissue typing. *Hum Fertil (Camb)*. 2005;8(1):5-11. doi:10.1080/14647270500030597

135. Bahadur G. Parliamentary proposals for liberal approaches to assisted conception. *Reproductive Biomedicine Online*. 2005;11(2):177-182.

136. Bahadur G. Concerns of sex selection and regulation in the report on Human Reproductive Technologies and the Law. *Reprod Biomed Online*. 2005;11(1):13-14. doi:10.1016/s1472-6483(10)61290-4

137. Sex selection and preimplantation genetic diagnosis. *Fertility and Sterility*. 2004;82:245-248. doi:10.1016/j.fertnstert.2004.05.016

138. Final report of the US President’s Council on Bioethics and Assisted Conception. *Reprod Biomed Online*. 2004;8(6):648.

139. Wolfson A. Getting serious about IVF. *New Atlantis (Washington, DC)*. 2004;5:78-84.

140. Williamson S. Sex (IST) selection? *Medical Law International*. 2004;6(3):185-206.

141. Williams N. “Designer” babies. *Current biology : CB*. 2004;14(15).

142. Westphal SP. The rush to pick a perfect embryo. *New Scientist*. 2004;182(2451):6-7.

143. Vastag B. Merits of Embryo Screening Debated. *Journal of the American Medical Association*. 2004;291(8):927-929.

144. Spriggs M. Commodification of children again and non-disclosure preimplantation genetic diagnosis for Huntington’s disease. *Journal of Medical Ethics*. 2004;30(6):538.

145. Sheldon S, Wilkinson S. Hashmi and Whitaker: an unjustifiable and misguided distinction? *Medical Law Review*. 2004;12(2):137-163.

146. Robertson JA. Reproductive technology in Germany and the United States: An essay in comparative law and bioethics. *Columbia Journal of Transnational Law*. 2004;43(1):189-227.

147. Robertson JA. Protecting embryos and burdening women: assisted reproduction in Italy. *Human Reproduction*. 2004;19(8):1693-1696.

148. Robertson JA. Gender variety as a valid choice: a comment on the HFEA - response to Edgar Dahl’s “The presumption in favour of liberty.” *Reproductive Biomedicine Online*. 2004;8(3):270-271.

149. Rao R. Preimplantation genetic diagnosis and reproductive equality. *Gender Medicine*. 2004;1(2):64-69.

150. Ram N. Britain permits controversial genetic test. *Hastings Center Report*. 2004;34(5).

151. Plachot M, Cohen J. Regulations for Preimplantation Genetic Diagnosis in France. *Journal of Assisted Reproduction and Genetics*. 2004;21(1):5-6.

152. Pennings G. Sex selection, public policy and the HFEA’s role in political decision making - response to Edgar Dahl’s “The presumption in favour of liberty.” *Reproductive Biomedicine Online*. 2004;8(3):268-269.

153. Munne S, Cohen J. The status of preimplantation genetic diagnosis in Japan: a criticism. *Reproductive Biomedicine Online*. 2004;9(3):258-259.

154. Mulvenna B. Pre-implantation genetic diagnosis, tissue typing and beyond: the legal implications of the Hashmi case. *Medical Law International*. 2004;6(2):163-182.

155. Menezo YJ, Lichtblau K, Elder K, Viville S, Dale B. Preimplantation genetic diagnosis (PGD) in France. *Journal of Assisted Reproduction & Genetics*. 2004;21(1):7-9.

156. Krones T, Richter G. Preimplantation genetic diagnosis (PGD): European perspectives and the German situation. *Journal of Medicine and Philosophy*. 2004;29(5):623-640.

157. Knoppers BM, Isasi RM. Regulatory approaches to reproductive genetic testing. *Human Reproduction*. 2004;19(12):2695-2701.

158. Kahn JP, Mastroianni AC. Creating a stem cell donor: A case study in reproductive genetics. *Kennedy Institute of Ethics Journal*. 2004;14(1):81-96.

159. Fost NC. Conception for donation. *JAMA*. 2004;291(17):2125-2126. doi:10.1001/jama.291.17.2125

160. Deffieux X. [Assisted reproductive technologies. Clinical and biological aspects and ethical considerations]. *Rev Prat*. 2004;54(12):1375-1382.

161. Darnovsky M. Revisiting sex selection: the growing popularity of new sex selection methods revives an old debate. *Genewatch*. 2004;17(1):3-6.

162. Dahl E. The presumption in favour of liberty: a comment on the HFEA’s public consultation on sex selection. *Reproductive BioMedicine Online*. 2004;8(3):266-267. doi:10.1016/S1472-6483(10)60906-6

163. Caulfield T, Knowles L, Meslin EM. Law and policy in the era of reproductive genetics. *Journal of Medical Ethics*. 2004;30(4):414-417. doi:10.1136/jme.2002.001370

164. Brownsword R. Reproductive opportunities and regulatory challenges. *Mod Law Rev*. 2004;67(2):304-321. doi:10.1111/j.1468-2230.2004.00488.x

165. Bosch X. UK criticized for embryo screening decision. *Nature Medicine*. 2004;10(12):1266-1266. doi:10.1038/nm1204-1266b

166. Blackburn E, Rowley J. Reason as Our Guide. *PLOS Biology*. 2004;2(4):1-1.

167. Bernal SK. Bioethics and Law Forum*: Ethical Offspring? *Journal of Andrology*. 2004;25(5):667-670. doi:10.1002/j.1939-4640.2004.tb02838.x

168. Benner P. The dangers of geneticism. *J Midwifery Womens Health*. 2004;49(3):260-262. doi:10.1016/j.jmwh.2004.02.012

169. Wasserman D. Having one child to save another: a tale of two families. *Philosophy & public policy quarterly / the Institute for Philosophy and Public Policy, School of Public Affairs, University of Maryland*. 2003;23(1-2):21-27.

170. Robertson JA. Procreative liberty in the era of genomics. *American Journal of Law and Medicine*. 2003;29(4):439-487.

171. Robertson JA. Extending preimplantation genetic diagnosis: medical and non-medical uses. *Journal of Medical Ethics*. 2003;29(4):213-216.

172. Plummer KM. Ending parents’ unlimited power to choose: legislation is necessary to prohibit parents’ selection of their children’s sex and characteristics. *Saint Louis University law journal*. 2003;47(2):517-560.

173. Nerlich B, Clarke A, Dingwall R. The first ‘designer baby’: The role of narratives, clichés and metaphors in the year 2000 media debate. *Science as Culture*. 2003;12(4):471-498.

174. Magnusson RS. A short history of the next thirty years: genetic testing, clinical care and personal choices. *The University of New South Wales law journal*. 2003;26(3):743-754.

175. Lohmann G. On the relation between moral, legal and evaluative justifications of pre-implantation genetic diagnosis (PGD). *Ethical perspectives / Catholic University of Leuven ; European Centre for Christian Ethics*. 2003;10(3-4):196-203.

176. Laing J. In vitro fertilisation, “treatment services” and embryo “suitability”. R (on the application of Quintavalle) v. Human Fertilisation and Embryology Authority. *Medical Law Review*. 2003;11(2):241-246.

177. King D. Why we should not permit embryos to be selected as tissue donors. *Bulletin of Medical Ethics*. 2003;(190):13-16.

178. Hunt J, Afnan M. Sex selection: Choice and responsibility in human reproduction. Response of the British Fertility Society to the public consultation document. *Human Fertility*. 2003;6(1):6-8.

179. Herissone-Kelly P. Bioethics in the United Kingdom: Genetic screening, disability rights, and the erosion of trust. *Cambridge Quarterly of Healthcare Ethics*. 2003;12(3):235-241.

180. Godard B, ten Kate L, Evers-Kiebooms G, Aymé S. Population genetic screening programmes: principles, techniques, practices, and policies. *Eur J Hum Genet*. 2003;11(2):S49-S87. doi:10.1038/sj.ejhg.5201113

181. Gillott J. “Reprogenetics”: hype, phobia and choice: an unreserved advocate of new reproductive technologies argues his case. *Conscience*. 2003;24(4):10-14.

182. Chneiweiss H. Sur les sentiers escarpés des montagnes de bioéthique : épisode 3: aux confins de l’eugénisme. *ms*. 2003;19(5):634-635.

183. Afnan M, Hunt J. Sex selection: The response of the British Fertility Society to the Human Fertilisation and Embryology Authority consultation document. *Human Fertility*. 2003;6(1):3-5. doi:10.1080/1464770312331368893

184. Adams KE. Ethical considerations of applications of preimplantation genetic diagnosis in the United States. *Med Law*. 2003;22(3):489-494.

185. Spriggs M, Savulescu J. Saviour siblings. *Journal of Medical Ethics*. 2002;28(5):289.

186. Silva FG. Ethics of the new biology and genetic medicine (molecular ethics): Brief (re)view from the USA. *Pathology International*. 2002;52(9):555-562.

187. Shenfield F. Ethical issues in the genetic aspects of haemophilia. *Haemophilia*. 2002;8(3):268-272.

188. Shakespeare T. Birds, bees and laser beams: the idea of parents choosing the sex of their children is no longer science fiction. *New scientist (1971)*. 2002;176(2369):23.

189. Sermon. ESHRE Preimplantation Genetic Diagnosis Consortium: data collection III (May 2001). *Human Reproduction*. 2002;17(1):233-246.

190. Scott A. Legal responses to some of the new developments in reproductive technologies. Part 3: the future of reproductive technologies and the law. *Human Reproduction and Genetic Ethics*. 2002;8(2):24-28.

191. Robertson JA. Sex selection for gender variety by preimplantation genetic diagnosis. *Fertility and Sterility*. 2002;78(3):463.

192. Rakowski E. Who should pay for bad genes? *California Law Review*. 2002;90(5):1345-1414.

193. Orellana C. German ethics group advises against pre-implantation genetic diagnosis. *Lancet*. 2002;359(9321):1926.

194. Mori T, Watanabe H. Ethical considerations on indications for gender selection in Japan. *Journal of Assisted Reproduction and Genetics*. 2002;19(9):420-425.

195. Meseguer M, Alvarez J, Lopez AC, Saldana JL, Remohi J, Pellicer A. Gender selection: Ethical, scientific, legal, and practical issues. *Journal of Assisted Reproduction and Genetics*. 2002;19(9):443-446.

196. Farrell K. Where have all the young girls gone? Preconception gender selection in India and the United States. *Indiana Int Comp Law Rev*. 2002;13(1):253-281.

197. Adamson D. Regulation of assisted reproductive technologies in the United States. *Fertility and Sterility*. 2002;78(5):932-942. doi:10.1016/S0015-0282(02)04199-7

198. Matsuda I, Wada Y, Nishida T. Guidelines for genetic testing. The Japan Society of Human Genetics, Council Committee of Ethics. *Journal of human genetics*. 2001;46(3):163-165.

199. Ludwig M, Neuwinger J, Bauer O, Diedrich K. Preimplantation genetic diagnosis: the German situation. *Trends in Genetics*. 2001;17(8):473-474.

200. Kupker W, Schumann A, Klingelberger S, Diedrich K. Preimplantation genetic diagnosis in Germany: ethical responsibility and law. *Reproductive Biomedicine Online*. 2001;2(2):84-87.

201. Gottlieb S. US doctors say sex selection acceptable for non-medical reasons. *BMJ*. 2001;323(7317):828.

202. Ferriman A. UK approves preimplantation genetic screening technique. *BMJ*. 2001;323(7305):125.

203. Braude P. Preimplantation genetic diagnosis and embryo research--human developmental biology in clinical practice. *Int J Dev Biol*. 2001;45(3):607-611.

204. Ludwig M, Neuwinger J, Bauer O, Diedrich K. The situation of preimplantation genetic diagnosis in Germany: legal and ethical problems. *Prenatal Diagnosis*. 2000;20(7):567-570.

205. Jellinek MA. Disease prevention and the genetic revolution: defining a parental right to protect the bodily integrity of future children. *Hastings constitutional law quarterly*. 2000;27(2):369-395.

206. Saegusa A. Japanese university approves genetic tests on in vitro embryos. *Nature*. 1999;397(6719):461.

207. Golden F. Good Eggs, Bad Eggs. *Time*. Published online January 11, 1999. Accessed May 20, 2024. https://content.time.com/time/subscriber/article/0,33009,989984,00.html

208. Draper H, Chadwick R. Beware! Preimplantation genetic diagnosis may solve some old problems but it also raises new ones. *J Med Ethics*. 1999;25(2):114-120.

209. Viville S, Pergament D. Results of a survey of the legal status and attitudes towards preimplantation genetic diagnosis conducted in 13 different countries. *Prenatal Diagnosis*. 1998;18(13):1374-1380.

210. Viville S, Messaddeq N, Flori E, Gerlinger P. Preparing for preimplantation genetic diagnosis in France. *Human Reproduction*. 1998;13(4):1022-1029.

211. Verlinsky Y, Kuliev A. Progress in preimplantation genetics. *Journal of Assisted Reproduction and Genetics*. 1998;15(1):18-21.

212. Stein E. Choosing the sexual orientation of children. *Bioethics*. 1998;12(1):X-24.

213. Romeo-Casabona CM. Health and Eugenics practices: Looking towards the future. *European Journal of Health Law*. 1998;5(3):241-260.

214. Chadwick R, ten Have H, Husted J, Levitt M, McGleenan T, Shickle D et al. Genetic screening and ethics: European perspectives. *J Med Philos*. 1998;23(3):255-273. doi:10.1076/jmep.23.3.255.2580

215. Botkin JR. Ethical issues and practical problems in preimplantation genetic diagnosis. *J Law Med Ethics*. 1998;26(1):17-28, 3. doi:10.1111/j.1748-720x.1998.tb01902.x

216. Bastijn SE. Preimplantation genetic diagnosis (PGD) in a European context. *Biomed Ethics*. 1998;3(3):85-89.
